# Bayesian stock assessment of Pacific herring in Prince William Sound, Alaska

**DOI:** 10.1371/journal.pone.0172153

**Published:** 2017-02-21

**Authors:** Melissa L. Muradian, Trevor A. Branch, Steven D. Moffitt, Peter-John F. Hulson

**Affiliations:** 1 Quantitative Ecology and Resource Management, University of Washington, Seattle, Washington, United States of America; 2 School of Aquatic and Fishery Sciences, University of Washington, Seattle, Washington, United States of America; 3 Alaska Department of Fish and Game, Cordova, Alaska, United States of America; 4 Alaska Fisheries Science Center, National Oceanic and Atmospheric Administration, Juneau, Alaska, United States of America; Sveriges lantbruksuniversitet, SWEDEN

## Abstract

The Pacific herring (*Clupea pallasii*) population in Prince William Sound, Alaska crashed in 1993 and has yet to recover, affecting food web dynamics in the Sound and impacting Alaskan communities. To help researchers design and implement the most effective monitoring, management, and recovery programs, a Bayesian assessment of Prince William Sound herring was developed by reformulating the current model used by the Alaska Department of Fish and Game. The Bayesian model estimated pre-fishery spawning biomass of herring age-3 and older in 2013 to be a median of 19,410 mt (95% credibility interval 12,150–31,740 mt), with a 54% probability that biomass in 2013 was below the management limit used to regulate fisheries in Prince William Sound. The main advantages of the Bayesian model are that it can more objectively weight different datasets and provide estimates of uncertainty for model parameters and outputs, unlike the weighted sum-of-squares used in the original model. In addition, the revised model could be used to manage herring stocks with a decision rule that considers both stock status and the uncertainty in stock status.

## Introduction

Lower-trophic level species form an integral part of marine food web dynamics by transferring production from plankton to larger piscivorous species [1,2]. Globally, low-trophic level species, such as sardine, herring, and sand lance, account for more than 30% of fisheries production [2]. Therefore maintaining the abundance of small pelagic “forage” fish is inextricably tied to both ecological and anthropocentric food security.

**Fig 1:**
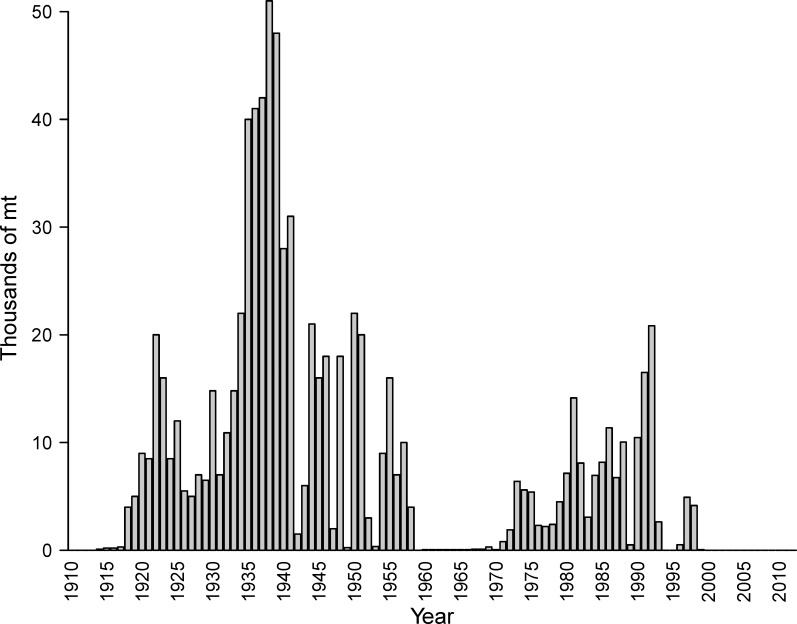
Commercial harvests of Pacific herring reported for Prince William Sound, 1914 through 2012 [5].

Pacific herring (*Clupea pallasii*) are a key pelagic forage fish in Prince William Sound (PWS), Alaska. In the Sound, the herring population provides valuable prey for marine birds, marine mammals such as sea lions and humpback whales, and piscivorous fish such as pink salmon and Pacific cod [3,4]. Humans have harvested Pacific herring for thousands of years and PWS herring have been commercially harvested for over a century [5,6]. Historical records indicate this population sustained a catch of over 40,000 mt for five consecutive years in the 1930s (Fig 1). Therefore, herring in PWS are important components of the ecosystem and were historically an important part of the local economy.

In 1989, PWS was the site of the *Exxon Valdez* oil spill, which occurred at the end of March, during the herring-spawn season that year. The spill occurred when the eponymous oil tanker ran aground off Bligh Island, spilling millions of gallons of crude oil into the Sound, and soiling over 1,000 miles of Alaskan coastline. Each spring, hundreds of millions of herring migrate from more open areas to shallower, coastal waters of PWS to spawn [7], thus herring were adversely affected by the spill in 1989 and the following year [8,9,10,11]. During their pre-spawn activity, herring form aggregations of millions of fish. This behavior incidentally makes them easy targets for predators within the Sound and attracts predators from within and outside PWS [4]. For example, humpback whales migrate into the Sound to prey on the pre-spawn herring aggregations [12]. Therefore, increased species diversity coincides with the herring-spawn, and as a result, many permanent and transitory species experienced negative effects from the *Exxon Valdez* oil spill [13,14,15].

**Fig 2:**
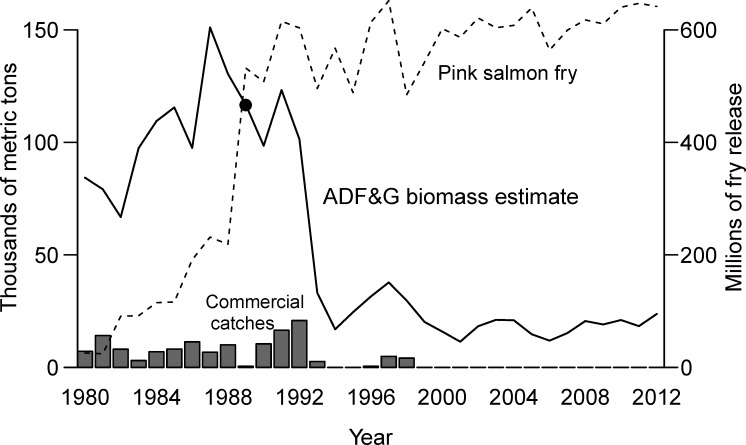
Alaska Department of Fish and Game estimates of pre-fishery run biomass (management metric used to set catch allowance) with bar plots of aggregated commercial catch data from Prince William Sound over the modeling horizon (1980–2012). Both quantities are on the same scale of thousands of metric tons (left axis). The dot denotes 1989, the year of the *Exxon Valdez* Oil Spill. The thin, black, dashed line shows numbers in millions (right axis) of hatchery salmon fry released into rivers that feed into the Sound.

In the years immediately following the spill, total herring biomass remained high in PWS until the population collapsed in 1993 [16]. No commercial harvest of herring occurred during the spring of 1989 because the spill occurred just before the commercial fishery spring season began that year. However, commercial fishing resumed in fall 1989. Over 10,000 mt of total catch was taken in 1990, and total catch in 1992 was higher than in any of the previous thirty-five years of fishing (Fig 1). Herring were expected to have record high recruits in 1993, but harvest was limited in 1993 due to extremely low observed spawning biomass [16,17,18]. In retrospect, 1993 marked the historic collapse of the herring population that underwent years of causal investigation. Herring commercial fisheries were closed in 1994, 1995, and the spring of 1996 due to low biomass, opened again in the fall of 1996–1998 due to trends of increasing biomass, and closed once more in 1999 due to insufficient biomass (Figs 1 and 2). Since 1999, the Sound has been closed to commercial herring fishing due to continued low abundance, which has cost Alaskan communities approximately $230 million dollars in lost income [19].

Once it was clear that herring were not recovering from the collapse in 1993, research programs were initiated to intensively monitor the post-spill abundance and body condition of herring, as well as collect data on physical and environmental factors to guide restoration efforts. While recovery of this population is important in its own right, allowing it to rebuild has become a focus of the overall restoration efforts in the Sound due to herring’s important role in that ecosystem’s food web dynamics; the hope is that a herring recovery will assist in rebuilding many other non-recovering species affected by the *Exxon Valdez* oil spill.

### Managing the herring fisheries in the Prince William Sound

The Alaska Department of Fish and Game (ADF&G) has managed PWS herring since 1960. Currently, they collect data on herring and use an assessment model to forecast pre-fishery run biomass. This forecast quantity is the projected biomass at the start of the spring fishing season, and is used to set harvest rates using a lower and an upper regulatory biomass threshold. If pre-fishery run biomass falls below the lower regulatory threshold of 22,000 short tons (19,958 mt), then the fishery is closed that year. The lower regulatory threshold is based on a minimum spawning biomass threshold of 25% of the potential spawning biomass from an un-fished state [20] using methods similar to those described in Funk and Rowell [21]. If forecasted biomass is between the lower regulatory threshold and an upper regulatory threshold of 42,500 short tons (38,555 mt), then the harvest rate may be set between 0.0 yr^-1^ and 0.2 yr^-1^; and if forecasted biomass is above the upper regulatory threshold, then the harvest rate may be set to 0.2 yr^-1^. The guideline harvest level is then divided based on the regulatory management plan among the five herring fisheries: the gillnet sac roe fishery, the pound spawn-on-kelp fishery, the purse-seine sac roe fishery, the food/bait fishery, and the spawn-on-kelp not in pounds fishery. These primary herring fisheries are described in the next paragraph.

**Fig 3:**
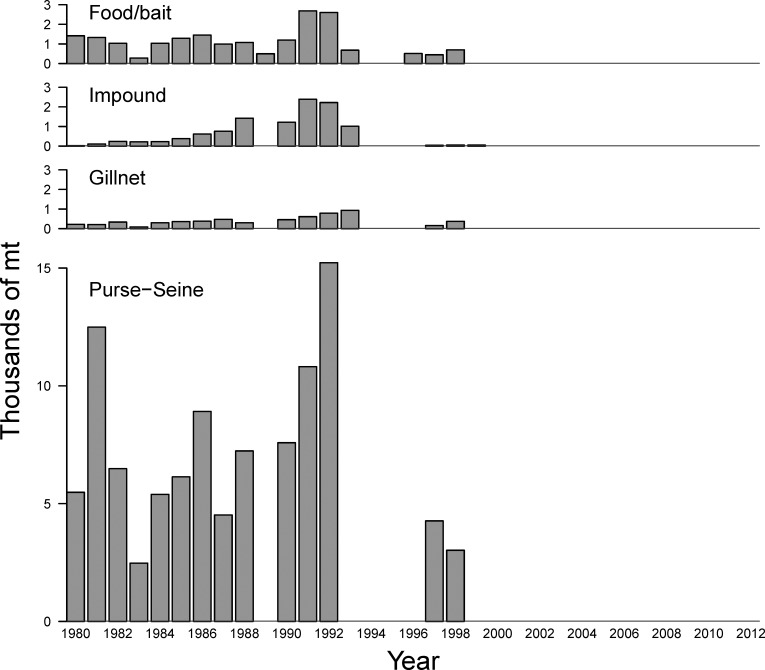
The four types of annual catch data, in thousands of metric tons, for Prince William Sound herring used in the Bayesian age-structured assessment model. Data for the three fisheries in the top panels are in the form of numbers of catch-at-age, so these were converted to annual total yield in mt using the weight-at-age (mt) data (1.7) for ease of display. Absent bars denote years that fishery did not run; all herring fisheries have been closed since 1999.

**Fig 4:**
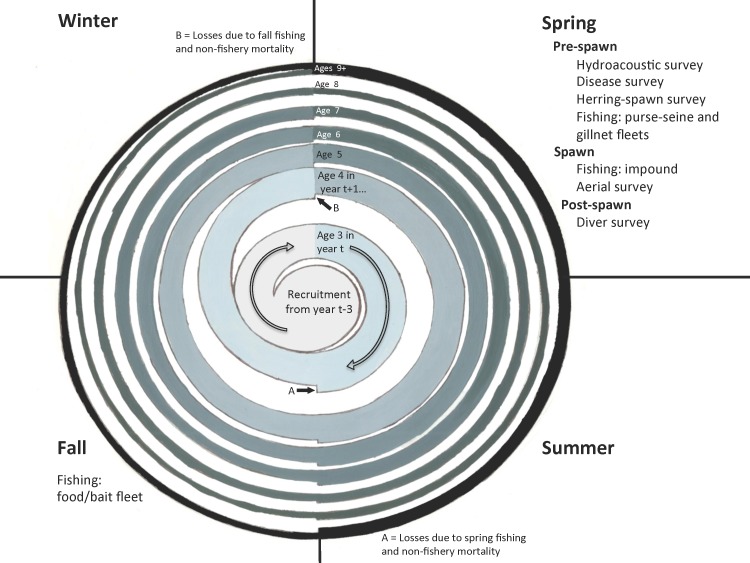
A schematic of the seasonal timing of fishing and sampling events included in the assessment model along with a schematic of a single cohort over seven years. Starting in the center of the spiral, the width of each separately colored curl represents the relative size of the cohort at a certain age and lighter colors denote younger ages of the cohort in earlier years. The cohort is reduced by fishery and non-fishery mortality effects (in that order) after the first 6 months (event A) and the last 6 months (event B) of every year before becoming a year older. The plus group is represented as a complete circle with two inputs: herring of age 8 and herring already in the plus group.

The food/bait fishery was conducted during the fall, and almost exclusively used purse-seine gear (and rarely trawl gear) to harvest whole herring for use as bait on hook and line gear or for human consumption (Figs 3 and 4). The remaining four fisheries occurred during the spawning season in the spring (Figs 3 and 4). The purse-seine sac-roe fishery harvested herring for egg or roe sacs using purse seine and gillnets and was actively managed to obtain the highest valued product possible through monitoring of ripening females, for body size and mature roe percentage, and timing of the main spawning event. During spawning, females deposit their eggs on marine vegetation in the inter-tidal zone, and these egg-encrusted kelp fronds are harvested by spawn-on-kelp fisheries [7,22]. The PWS pound fishery is a type of spawn-on-kelp fishery that involves impounding mature herring in a net with suspended kelp, until they spawned. Herring that survive being impounded are released and the kelp with eggs attached, called komochi konbu, is harvested and sold.

### Past development of the assessment model and a brief description of key assumptions and data

Stock assessment is the practice of fitting a model to data to estimate stock status by using an assumed statistical relationship between predicted and observed values. In fisheries management, assessment models are used to estimate life-history parameters needed to set catch limits or harvest rates based on the size and productivity of exploited populations. Therefore, accurate assessment model estimates are necessary to prevent overfishing or underutilization of a managed stock.

Beginning in 1973, ADF&G estimated PWS herring biomass using data from an aerial survey of male spawning biomass until 1988, when an age-structured assessment model (known as the ASA Model) was developed. This original ASA Model has been expanded and updated here to incorporate more data types, in addition to the changes noted, and further changes are described below. The initial version of the ASA Model used catch-at-age data, along with the aerial milt survey information, but also incorporated annual numbers of eggs deposited as an absolute index of female spawning biomass [5]. Later, an assumption of increased levels of mortality from disease starting in 1992 was incorporated into the ASA model to fit observed trends in the population [16,23]. An index of hydroacoustic biomass was formally incorporated into the ASA model to resolve a conflicting trend between the mile-days of milt and egg-deposition data over 1987–91 by adding information to the model that emphasized one of the conflicting trends over the other [17]. Even though evidence is lacking for a Ricker stock-recruit relationship for PWS herring, this relationship was introduced to the ASA model to stabilize estimates of recruitment [17]. The assumed Ricker relationship operates as a penalty to keep estimated age-3 abundance close to the assumed recruitment relationship and to avoid recruitment from converging towards zero in some years. Further details of the PWS herring ASA Model used by ADF&G are included in the Materials and Methods.

### Challenges to modeling the population dynamics of Prince William Sound herring

A number of competing hypotheses have been proposed to explain the recent population trends of PWS herring. Proposed explanations related to direct and indirect effects from the oil spill include: toxicity related to ingestion of hydrocarbons, recruitment failure related to increased egg loss, and poor over-wintering condition related to poor growth and low plankton levels [17,24]. Some debate exists as to whether these factors caused the herring collapse of 1992–93, and even more speculation surrounds the causes of the continued low abundance. Potential explanations of the continued low abundance include persistent effects of the oil spill, increased predation coinciding with increasing populations of humpback whales [12], increased disease mortality, recruitment failure, and increased interspecific competition with introduced pink salmon [24]. In relation to the latter potential factor, hatchery pink salmon fry releases in PWS more than doubled to over 500 million fry between 1985 and 1988 (Fig 2). Juvenile pink salmon compete with herring for food, and eat age-0 and age-1 herring [24]. The former interaction is of particular importance because Pacific herring show evidence of density-dependent growth [25], meaning limited food resources can reduce fat stores, leading to increased over-winter mortality. To test hypotheses about factors influencing population status, assessment scientists need the best available tools to reliably estimate population metrics.

Several statistical frameworks exist to implement a population dynamics model as a stock assessment tool, and Bayesian methods offer several advantages beyond frequentist and maximum likelihood methods. The ASA model currently employs a weighted least squares estimation framework that provides point estimates of the forecast quantity (Fig 2), and thus ADF&G does not formally incorporate variance into their decision rule. Furthermore, the weighting does not utilize estimates of sampling error (when provided), but is chosen to reflect consensus views about the relative precision of each dataset [17]. In contrast, maximum likelihood methods replace subjective weights with theoretically valid estimates of uncertainty based on the assumed form of the distribution. Bayesian methods incorporate this improvement and offer further advantages, such as easily interpretable estimates of variance in the form of probability distributions (posteriors), [26,27,28]. Furthermore, Bayesian methods can incorporate the full range of uncertainty across models and parameters, in contrast to conditional maximum likelihood methods that may fail to incorporate the actual variability of several parameters [27,29]. In addition to using information contained in the data, Bayesian theory allows formal incorporation of expert opinion, or information from other stocks or species, into the model fitting process through the use of prior probability distributions. Therefore, in addition to updating the PWS herring ASA by testing assumptions and investigating evidence of a retrospective pattern, this study implements the model in a Bayesian framework to provide the following benefits: 1) a conceptually simple definition of uncertainty via credible intervals, and 2) the assignment of probabilities to hypotheses concerning population dynamics and state of the fishery.

## Materials and methods

Fifteen data types were used in this study (Table 1), including weight-at-age, age compositions, a milt index, a hydroacoustic biomass index from the ADF&G surveys, and a biomass index from the Prince William Sound Science Center (PWSSC) hydroacoustic survey. Note that equations are listed in the tables and referred to using equation indices, for example Equation 1.1 will be the first equation in Table 1. The model starts in 1980, since this is the start of the longest time series of biomass data (1980–2012), but many series have years or sets of years of missing data (Table 1). We developed the Bayesian form of the ASA model in AD Model Builder [30]. In short, MCMC based Bayesian modeling begins by sampling a set of parameter values from the specified prior distributions then uses the model to compute the likelihood of the observed data given the set of sampled parameter values. This information is used to update the estimate of each parameter using Bayes’ formula. Then the MCMC algorithm repeats this process many times to obtain a distribution of parameter estimates (the posterior distribution), which characterize each parameter’s variability. Using a single MCMC chain, posterior distributions were obtained for all parameters and key model outputs, such as pre-fishery run biomass and fishing mortality. The following sections describe the data, the population dynamics and modeling assumptions, the forms of the likelihood functions and prior distributions, and convergence criteria used when fitting the Bayesian ASA model.

**Table 1:** Time series used in the Bayesian ASA model. The first column lists the index number used in the text, the next columns list the data type and units, n_y_ refers to number of years that data type was collected, and the final column reports the first and last year of collection. Note some series are discontinuous.

Index	Data type	Units	Symbol	n_y_	Years
1.1	Gillnet catch-at-age	millions	*C*_2,*y*,*a*_	15	(1980, 1998)
1.2	Pound utilization catch-at-age	millions	*C*_3,*y*,*a*_	16	(1980, 1999)
1.3	Food/bait catch-at-age	millions	*C*_4,*y*,*a*_	17	(1980, 1998)
1.4	Disease index of VHSV prevalence, ages 3–4	percentage	*d*_1,*y*_	19	(1994, 2012)
1.5	Disease index of *I*. *hoferi* prevalence, ages 5–8	percentage	*d*_2,*y*_	19	(1994, 2012)
1.6	Fecundity-at-age	no. of eggs per female	*f*_*y*,*a*_	7	(1984, 1993)
1.7	Weight-at-age of spawning herring	mt/million fish	*w*_*y*,*a*_	33	(1980, 2012)
1.8	Purse-seine age-composition	proportion	*Θ*_1,*y*,*a*_	13	(1980, 1998)
1.9	Spawner survey age-composition	proportion	*Θ*_*Sp*,*y*,*a*_	31	(1982, 2012)
1.10	Female spawners	proportion	*ρ*_*f*,*y*_	33	(1980, 2012)
1.11	Total annual purse-seine yield	mt	Ω_1,*y*_	13	(1980, 1998)
1.12	Eggs deposited	trillions	*E*_*y*_	10	(1984, 1997)
1.13	C.V. for eggs deposited		*σ*_*E*,*A*,*y*_	10	(1984, 1997)
1.14	ADF&G hydroacoustic survey biomass	mt	*H*_1,*y*_	5	(2005, 2009)
1.15	PWSSC hydroacoustic survey biomass	mt	*H*_2,*y*_	20	(1993, 2012)
1.16	C.V. for PWSSC hydroacoustic biomass		$\sigma_{H_{2,A,y}}$	20	(1993, 2012)
1.17	Milt	mile/day	*T*_*y*_	33	(1980, 2012)

### Fishery-dependent data

Catch data over the period 1980–1999 from the four major herring-fishing fleets in the Sound were used (Fig 3). Catch-at-age data (in millions of fish) came from the gillnet sac roe fishery (Eq 1.1), the pound-utilization spawn-on-kelp fishery (Eq 1.2), and the food/bait fishery (Eq 1.3). The food/bait fishery was conducted during the fall, and the former two fisheries ran during the spawning season in the spring (Fig 4). Based on information in [17], we assume that 75% of impounded fish die during the spawn-on-kelp fishery, and set *ρ*_*k*_ = 0.75 (Eq 2.2, Table 2). Annual data from the purse seine sac roe fishery is in the form of total yield, in metric tons, and catch at-age proportions (Eq 1.8, 1.11).

**Table 2:** Model formulation, first column gives the equation number, the second column gives a description, and the final column gives the mathematical form of the dynamics.

No	Description	Equation
2.1	**Catch, millions of fish** Estimated total purse-seine catch	${\hat{C}}_{1,y}^{}=\frac{\Omega_{1,y}}{\sum_{a\in A}\left({\hat{\Theta }}_{1,y,a}w_{y,a}\right)}$
2.2	Spring removals, *ρ*_*k*_ = 0.75	${\hat{C}}_{S,y,a}={\hat{\Theta }}_{1,y,a}{\hat{C}}_{1,y}+C_{2,y,a}+\rho_{k}C_{3,y,a}$
	**Survival, rate**	
2.3	Half-year survival, 1980–1991, all ages	$S_{y,a}^{.5}=e^{-.5m_{a}}$
2.4	Half-year survival, 1993–2012, ages 3–4, with VHSV mortality	$S_{y,a}^{.5}=e^{-.5{(m}_{a}+\beta_{1}d_{1,y})}$
2.5	Half-year survival, 1993–2012, ages 5–8, with *I*. *hoferi* mortality, collection era *x = 1*,*2*	$S_{y,a}^{.5}=e^{-.5(m_{a}+\beta_{2,x}d_{2,y})}$
2.6	Half-year survival, 1993–2012, plus group	$S_{y,9^{+}}^{.5}=S_{y-1,9^{+}}^{.5}\left(\frac{S_{y,8}^{.5}}{S_{y-1,8}^{.5}}\right)$
2.7	**Selectivity, logistic form** Purse-seine gear selectivity by age	$V_{a}=\frac{1}{1+e^{-\beta_{v}(a-\alpha_{v})}}$
	**Abundance, millions of fish**	
2.8	Pre-fishery total abundance, ages 4–8	$N_{y+1,a+1}=\left[((N_{y,a}-{\hat{C}}_{S,y,a})S_{y,a}^{.5})-C_{4,y,a}\right]S_{y,a}^{.5}$
2.9	Pre-fishery total abundance, ages 9^+^	$N_{y+1,9^{+}}=\left[((N_{y,8}-{\hat{C}}_{S,y,8})S_{y,8}^{.5})-C_{4,y,8}\right]S_{y,8}^{.5}$+
		$\left[(N_{y,9^{+}}-{\hat{C}}_{S,y,9^{+}})S_{y,9^{+}}^{.5}-C_{4,y,9^{+}}\right]S_{y,9^{+}}^{.5}$
2.10	Post-fishery spawning abundance	${\tilde{N}}_{y,a}=\rho_{M,a}\left[N_{y,a}-({\hat{\Theta }}_{1,y,a}{\hat{C}}_{1,y}+C_{2,y,a}+C_{3,y,a})\right]$
	**Biomass, mt**	
2.11	Pre-fishery total biomass	$B_{y}=\sum_{a\in A}\left(N_{y,a}w_{y,a}\right)$
2.12	Pre-fishery spawning biomass	${\tilde{B}}_{y}=\sum_{a\in A}\rho_{M,a}N_{y,a}w_{y,a}$
2.13	Post-fishery spawning biomass	${\tilde{B}}_{post,y}=\sum_{a\in A}{\tilde{N}}_{y,a}w_{y,a}$
2.14	Estimated 2013 pre-fishery run biomass	$B_{2013}=B_{2013,3}+\sum_{a\in A_{-3}}\rho_{M,a}N_{2013,a}{\bar{w}}_{a}$
2.15	Average weight-at-age over the last 5 years	${\bar{w}}_{a}=\frac{1}{5}\sum_{i=2008}^{2012}w_{i,a}$
2.16	Estimated 2013 age-3 biomass	$B_{2013,3}=\rho_{M,2,3}{\bar{w}}_{3}exp\left(\frac{1}{10}\sum_{i=2003}^{2012}ln(\eta_{i})\right)$
	**Estimates used in the likelihood expressions**	
2.17	Estimated ADF&G hydro-acoustic biomass, mt	${\hat{H}}_{1,y}=B_{y}e^{q_{1}}$
2.18	Estimated PWSSC hydro-acoustic biomass, mt	${\hat{H}}_{2,y}=B_{y}e^{q_{2}}$
2.19	Estimated purse-seine age composition	${\hat{\Theta }}_{1,y,a}=\frac{V_{a}N_{y,a}}{\sum_{a\in A}\left(V_{a}N_{y,a}\right)}$
2.20	Estimated spawning age composition	${\hat{\Theta }}_{Sp,y,a}=\frac{\rho_{M,a}N_{y,a}}{\sum_{a\in A}\left(\rho_{M,a}N_{y,a}\right)}$
2.21	Estimated naturally spawned eggs, trillions	${\hat{E}}_{y}=10^{-6}\rho_{f,y}\sum_{a\in A}({\tilde{N}}_{y,a}f_{y,a})\;\forall \;y\in Y$
2.22	Estimated milt, mile-days	${\hat{T}}_{y}=\frac{(1-\rho_{f,y}){\tilde{B}}_{post,y}}{e^{q_{T}}}$

### Fishery-independent data

Fishery-independent surveys include a hydroacoustic survey conducted by PWSSC and several surveys conducted by ADF&G during the herring-spawn (described in the following paragraphs) (Fig 4). ADF&G uses purse-seine and cast net gear to sample spawning assemblages and collect information on age compositions (Eq 1.9), weight-at-age (Eq 1.7), and the proportion of spawning herring that are female (Eq 1.10).

Two separate indices of hydroacoustic abundance are used in the model; one collected by the PWSSC and one collected by ADF&G (Eq 1.14–1.15). The PWSSC began collecting herring hydroacoustic biomass data in 1993. The first two annual surveys were conducted each fall and the remaining surveys occurred during the herring spawn each spring. As input into the ASA Model these two PWSSC series were combined into a single time series [18]. Furthermore, this study includes only those ADF&G observations that are independent from the PWSSC survey (Eq 1.14). Even though ADF&G began conducting acoustic surveys in 1997, data used by ADF&G were a combination of their observations and those of the PWSSC until 2004 [4,17]. Independent ADF&G hydroacoustic biomass estimates are available for years 2005–2009.

The egg deposition index is based on a combination of ADF&G purse seine data from 1984 and 1988–1992 that collected herring used to measure herring fecundity-at-age (Eq 1.6), and dive surveys in 1984, 1988–1992, and 1994–1997 to estimate the total numbers of eggs deposited in the inter-tidal spawning beds (Eq 1.12). We converted the 95% confidence intervals from the egg deposition data into coefficients of variation (Eq 1.13). There is high uncertainty associated with this index due to inter-annual changes in spawning location within the Sound and because egg loss, due to wave-action and predation, occurs during any lag between the diver survey and the spawning event [31]. We therefore estimate additional error in egg deposition to account for this combination of process and measurement error (Eq 3.14, Table 3). In the model, a key assumption is that egg deposition is directly related to absolute spawning biomass after accounting for fecundity-at-age data and numbers-at-age estimates (Eq 2.21).

**Table 3:** Key model parameter estimates (medians and 95% credible intervals, CI). All mortality is modeled as instantaneous mortality rates.

Index	Parameters	Symbols	Parameter values Median and 95% interval		Prior
3.1	Background mortality, 1980–91, ages 3–8	*m*_*a*∈(3,…,8)_	0.25		Not estimated
3.2	Total mortality, 1980–91, age 9^+^	$m_{9^{+}}=\mu_{9^{+}}$	0.93	(0.60, 1.30)	*U*∼(0.30,2.00)
3.3	VHSV disease scalar, ages 3–4	*β*_1_	83.56	(18.18, 159.5)	*U*∼(0.00,1000)
3.4	*I*. *hoferi* scalar, ages 5–8, 1994–2006	*β*_2,1_	0.90	(0.33, 1.55)	*U*∼(0.00,25.00)
3.5	*I*. *hoferi* scalar, ages 5–8, 2007–12	*β*_2,2_	0.45	(0.03, 1.19)	*U*∼(0.00,25.00)
3.6	Disease mortality in 1993, VHSV	*m*_1,1993,*a*_ = *μ*_1,1993_	0.67	(0.16, 1.18)	*U*∼(0.00,5.00)
3.7	Disease mortality in 1993, *I*. *hoferi*	*m*_2,1993,*a*_ = *μ*_2,1993_	0.68	(0.23, 1.09)	*U*∼(0.00,5.00)
3.8	Purse-seine gear selectivity	*α*_*V*_	3.78	(3.49, 4.08)	*U*∼(3.00,5.00)
3.9	Purse-seine gear selectivity	*β*_*v*_	2.27	(1.60, 3.20)	*U*∼(1.00,7.00)
3.10	ADF&G acoustic scalar, log-link	*q*_1_	-0.36	(-0.79, 0.07)	*U*∼(−5.00,5.00)
3.11	ADF&G acoustic biomass CV	$\sigma_{H_{1}}$	0.29	(0.15, 0.55)	*U*∼(0.00,0.60)
3.12	PWSSC acoustic scalar, log-link	*q*_2_	-0.31	(-0.63, 0.01)	*U*∼(−5.00,5.00)
3.13	PWSSC acoustic biomass add’l error	$\sigma_{H_{2},B}$	0.34	(0.24, 0.52)	*U*∼(0.00,0.60)
3.14	Egg deposition additional error	*σ*_*E*,*B*_	0.40		Not estimated
3.15	Milt scalar, log-link	*q*_*T*_	322.58	(248.71, 421.78)	*U*∼(2.30,7.00)
3.16	Milt CV	*σ*_*T*_	0.33	(0.25, 0.44)	*U*∼(0.00,0.60)
3.17	Proportion mature at age 3, 1980–96	*ν*_3_; *ρ*_*M*,1,3_ = *ν*_3_*ρ*_*M*,1,4_	0.39	(0.28, 0.56)	*U*∼(0.00,0.75)
3.18	Proportion mature at age 4, 1980–96	*ρ*_*M*,1,4_	0.80	(0.62, 0.97)	*U*∼(0.00,1.00)
3.19	Proportion mature at age 3, 1997–2012	*ρ*_*M*,2,3_	0.49	(0.37, 0.66)	*U*∼(0.00,1.00)
3.20	Proportion mature at age 4, 1997–2012	*ρ*_*M*,2,4_	0.90		Not estimated
3.21	Recruitment by year (millions), log-link	*η*_*y*,3_ = ln(*N*_*y*,*a*_)	Table 4		*U*∼(0.00,8.01)
3.22	Age-4 abundance in 1980, log-link	*η*_1980,4_ = ln(*N*_1980,4_)	6.33	(6.10, 6.57)	*U*∼(0.00,8.01)
3.23	Age-5 abundance in 1980, log-link	*η*_1980,5_ = ln(*N*_1980,5_)	4.28	(3.88, 4.68)	*U*∼(0.00,8.01)

**Table 4:** Recruitment (median and 95% credible intervals) in millions of age-3 fish, pre-fishery run biomass (median and 95% interval) in 10^3^ mt, the probability that pre-fishery run biomass has fallen below the lower regulatory threshold (B<LRT), exploitation rate (median and 95% interval) defined as total catch in each year (converted to metric tons using the empirical weight-at-age matrix) divided by the 95% biomass trajectories (3^rd^ column), total instantaneous mortality for age 3–4 fish, and total instantaneous mortality for age 5–8 fish.

Year	Recruitment Median and 95% int.		Pre-fishery run biomass Median 95% int.		Prob. B<LRT	Exploitation rate Median 95% int.		Age 3–4 total mortality	Age 5–8 total mortality
1980	225.21	(163.41, 308.76)	57.23	(41.86, 76.58)	0.00	0.12	(0.09, 0.17)	0.25	0.25
1981	118.78	(78.67, 173.39)	66.26	(52.80, 85.69)	0.00	0.21	(0.17, 0.27)	0.25	0.25
1982	161.48	(112.27, 230.74)	57.70	(43.89, 77.93)	0.00	0.14	(0.10, 0.18)	0.25	0.25
1983	447.91	(340.35, 594.45)	67.50	(50.36, 91.18)	0.00	0.05	(0.03, 0.06)	0.25	0.25
1984	373.56	(279.42, 497.78)	79.52	(60.26, 106.03)	0.00	0.09	(0.07, 0.12)	0.25	0.25
1985	119.70	(79.73, 179.77)	96.28	(73.94, 126.78)	0.00	0.08	(0.06, 0.11)	0.25	0.25
1986	142.15	(96.18, 207.55)	84.73	(65.96, 110.90)	0.00	0.13	(0.10, 0.17)	0.25	0.25
1987	1237.93	(988.44, 1580.59)	95.60	(73.80, 126.20)	0.00	0.07	(0.05, 0.09)	0.25	0.25
1988	136.76	(94.92, 207.47)	122.03	(94.20, 158.26)	0.00	0.08	(0.06, 0.11)	0.25	0.25
1989	28.43	(18.86, 52.53)	124.99	(98.52, 161.09)	0.00	0.00	(0.00, 0.01)	0.25	0.25
1990	29.67	(11.85, 67.94)	107.57	(85.74, 138.74)	0.00	0.10	(0.08, 0.12)	0.25	0.25
1991	844.99	(566.63, 1287.31)	94.97	(74.38, 123.53)	0.00	0.17	(0.13, 0.22)	0.25	0.25
1992	62.41	(23.70 149.93)	93.74	(69.01, 129.80)	0.00	0.22	(0.16, 0.30)	0.93	0.94
1993	135.11	(65.92, 292.40)	38.83	(29.38, 51.21)	0.00	0.07	(0.05, 0.09)	0.93	0.94
1994	18.25	(6.92, 41.75)	20.49	(14.88, 27.92)	0.44	–		1.09	0.32
1995	94.46	(64.43, 136.20)	18.71	(14.10, 24.71)	0.67	–		0.37	0.36
1996	76.91	(47.93, 121.32)	20.52	(15.52, 27.02)	0.43	0.02	(0.02, 0.03)	0.25	0.36
1997	142.02	(81.34, 246.50)	27.92	(21.65, 36.78)	0.00	0.18	(0.13, 0.23)	0.33	0.35
1998	69.83	(39.03, 124.43)	21.89	(16.53, 29.38)	0.27	0.19	(0.14, 0.25)	0.73	0.39
1999	6.08	(1.4, 18.28)	14.93	(10.48, 21.09)	0.95	0.00	(0.00, 0.00)	0.26	0.36
2000	22.41	(10.23, 42.72)	13.43	(9.46, 18.70)	0.99	–		0.25	0.36
2001	10.27	(3.67, 22.91)	11.91	(8.42, 16.58)	1.00	–		0.26	0.44
2002	210.28	(145.21, 301.15)	14.66	(10.53, 20.46)	0.97	–		0.37	0.40
2003	39.97	(24.69, 63.64)	19.90	(14.48, 27.78)	0.51	–		0.26	0.54
2004	19.03	(10.25, 32.64)	20.33	(14.57, 28.42)	0.46	–		0.26	0.40
2005	26.15	(14.92, 44.22)	15.89	(11.26, 22.08)	0.92	–		0.26	0.40
2006	16.74	(8.77, 29.50)	13.70	(9.50, 19.34)	0.98	–		0.26	0.40
2007	102.28	(68.24, 150.81)	15.36	(10.83, 21.69)	0.94	–		0.25	0.38
2008	94.69	(62.78, 143.46)	21.14	(15.08, 29.76)	0.37	–		0.25	0.33
2009	28.11	(13.26, 55.63)	20.12	(14.36, 28.09)	0.48	–		0.25	0.34
2010	53.48	(22.39, 107.25)	20.58	(14.62, 28.91)	0.43	–		0.27	0.30
2011	9.23	(1.34, 48.71)	18.06	(12.53, 25.58)	0.72	–		0.25	0.32
2012	77.84	(18.94, 226.89)	18.14	(12.13, 26.66)	0.69	–		0.25	0.36
2013	35.29	(23.87, 52.75)	19.41	(12.15, 31.74)	0.54	–		–	–

The ADF&G aerial milt survey from 1980–2012 is the final dataset used (Eq 1.17). During spawning events, aerial surveys fly along the spawning sites and measure the linear extent of milt clouds in miles of corresponding coastline per day, hence the mile-days units. This metric was developed in 1987 to address the issue of residence uncertainty in the aerial biomass estimates historically used by the ADF&G to estimate spawning biomass [5]. Residence uncertainty refers to issues inherent in measuring peak spawning biomass using aerial surveys that require assumptions about the timing of fish movement in and out of the spawning beds during the weeks-long spawning period. Mile-days of milt are a key index of herring biomass that closely track the hydroacoustic biomass estimates.

**Fig 5:**
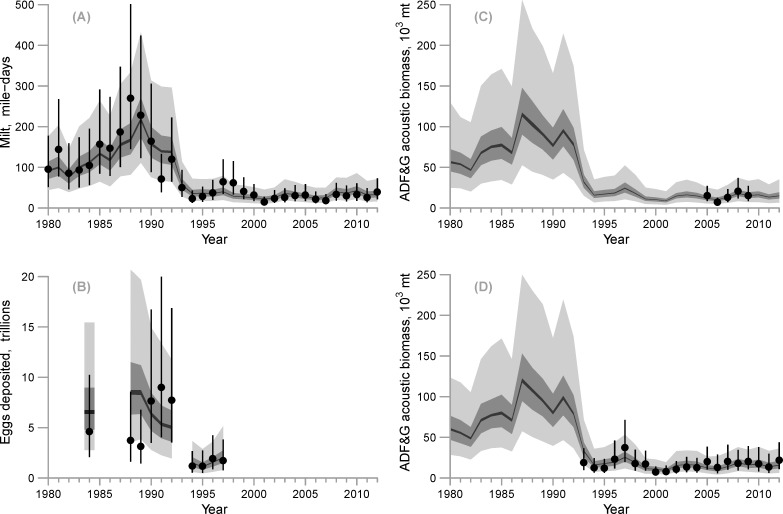
Model estimates and the four time series of abundance estimates (1980–2012): (A) mile-days of milt, (B) egg deposition surveys, (C) ADF&G hydroacoustic estimates, and (D) PWSSC hydroacoustic estimates. The solid circles and lines represent the mean and 95% confidence intervals of the data (plus additional variance estimated by the model); the shaded polygons represent the respective posterior predictive intervals (light gray = 95% interval, darker gray = 50% interval, black = 5% interval).

When the four indices of biomass (milt, egg deposition, and the two acoustic indices) are compared, complementary and conflicting trends can easily be identified (Fig 5). All indices display reasonably common trends after 1993. The indices of milt and egg deposition start out at relatively high levels during the late 1980s and drop to lower levels during the mid-1990s, but they show opposing trends during the years 1988–1992. Milt shows a tremendous increase that peaked in 1989 and then sharply decreased over the next six years (Fig 5A). This is in contrast to the trend in the egg deposition data, where moderate numbers of eggs were deposited in 1988–9, but numbers sharply increased over the next three years (Fig 5B). This is a well-documented conflict in the PWS herring data [17].

### Disease survey data

Infection data are included in the model for the protozoan parasite *Ichthyophonus hoferi* and the North American strain of viral hemorrhagic septicemia virus (VHSV), which are likely to have been present in PWS herring long before exploitation started. However, there were no reports of disease outbreaks before the late 1980s. Then, high levels of infection from *I*. *hoferi* were found during 1989–1993 and VHSV in 1993 [10,17,32,33]. These diseases were found to significantly affect herring mortality at the individual level [23]. This evidence combined with the recent population crash influenced the initialization of a systematic survey in 1994 to collect data on diseases affecting PWS herring. The model integrates this information as two series: the first is an index for *I*. *hoferi* infection (Eq 1.4–1.5) and the second is an index that combines direct evidence of VHSV and evidence of ulcers related to filamentous bacteria. Previous studies concluded that a combined VHSV/ulcer index better explains survival trends and fluctuations in associated annual mortality since ulcers can be a surrogate indicator of VHSV infection [32].

### Model formulation: Population dynamics, and prior distributions

The PWS herring population is managed as a fully mixed population under the assumption of no immigration or emigration from populations outside PWS because geographic barriers isolate PWS herring from other spawning populations [5]. Biomass losses occur due to either fishing events within the Sound (there is no evidence of fishing on this population outside of the Sound prior to 1980) or natural mortality (including disease), while increases occur through recruitment and growth in body weight with increasing age.

In common with the current ADF&G assessment model, the Bayesian model is a statistical catch-at-age model [34]. Three-year-old herring are the first age class that is observed to be present in large numbers on the spawning grounds and Pacific herring live up to fifteen years [35,36]. Therefore, age-3 and older fish are included in the model and a plus group containing herring of ages 9 and older is used to minimize the effects of ageing error [5]. Estimated parameters include the numbers at age for every age-class in the first year (1980) and recruitment at age 3 in the remaining 32 years (Eq 3.21–3.23). Unlike the current ADF&G model, the Bayesian model did not use the Ricker stock recruit relationship to keep estimated age-3 abundance positive, but instead used broad, positive uniform priors on the log-scale for recruitment, consistent with the assumption that herring recruitment is log-normally distributed. The remaining age-classes in 1980 were also estimated using broad, positive uniform priors in log-space. These parameters, along with fishery removals, natural mortality and gear selectivity parameters, supply information to complete a population matrix of pre-fishery total abundances for ages 3 to 9+ across years 1980–2012 (Eq 2.8–2.9).

Each model year begins at the start of the spawning season in the spring, and commercial fishing events occur in the spring and fall. For use in the pre-fishery abundance calculations, it was necessary to derive annual purse seine catch in millions (Eq 2.1) using observed purse-seine annual yield (mt), purse-seine proportion of catch at-age, and weight-at-age (mt) data (Eq 1.11, 1.8, 1.7). In common with the spring fisheries, all surveys are conducted during the pre-spawn and spawning period when herring form large aggregations and are easier to sample. The exception is the diver survey, which measures numbers of total herring eggs deposited in the Sound and therefore must be conducted after spawning is complete.

Two age-specific and time-independent instantaneous mortality rates were used to model biomass losses not attributed to fishing (predation, starvation, disease, etc.). One of these rates applies to herring of ages 3–8 and was assumed to remain constant at the value of 0.25 yr^-1^ prior to 1992 (Eq 3.1) because 0.25 yr^-1^ is thought to be the lowest realistic rate of average, instantaneous mortality for Pacific herring [16]. The other non-fishery mortality rate applies to fish in the plus group and was estimated using a uniform prior with bounds of (0.30 yr^-1^, 2.00 yr^-1^) (Eq 3.2), where the lower bound reflects the assumption that instantaneous mortality for the oldest fish in the population is higher than that of younger fish due to senescence, and the upper bound for this mortality was chosen to result in a sufficiently broad prior distribution. These rates are used to discretely model cumulative herring losses between fishing seasons (Eq 2.3–2.6) since fishing events occur in two periods and last for only a short time [5].

There is evidence of recent high levels of disease significantly affecting Pacific herring abundance [23]. Thus, additional mortality due to increased disease is added to the mortality rate for herring of ages 3–8 starting in model-year 1992 (Eq 2.4–2.5). The assumed functional form incorporates the assumption that disease mortality and survival are non-linearly related [17,32]. VHSV infection is assumed to increase the mortality rate for fish of ages 3–4, and *I*. *hoferi* infection to increase the mortality rate for fish of ages 5–8. These assumptions are based on infection prevalence rates for different ages in herring, albeit simplified from the complex relation between disease prevalence, mortality, and age. Estimated parameters scale each disease-infection index to mortality and can take on non-negative values with broad uniform priors (Eq 3.3–3.5). This study introduced separate *I*. *hoferi* scalars over 1994–2006 and 2007–2012 (Eq 3.4–3.5) to capture the potential difference in the relationship between the index and true mortality resulting from a change in detection method from histopathology to tissue explant culture.

One key assumption in the model is that additional mortality from disease began in 1992 even though the disease index begins in 1994. Thus, additional disease mortality in 1993 was estimated using broad, non-negative uniform priors (Eq 3.6–2.7), and mortality in the latter half of 1992 is set equal to that in 1993. This is important since the estimated additional mortality in 1992 and 1993 allows the model to account for the collapse in biomass that is evident in the milt and hydroacoustic indices of abundance [17,32]. Therefore, another way of thinking about “disease mortality” in these two years is that it is an estimate of the additional mortality from all sources (disease, predation, competition, oil-spill effects) required to explain the sharp decline in biomass over 1992–1993.

Gear selectivity is assumed to be a logistic function of age *a*, with two parameters, *α*_*v*_ and *β*_*v*_ (Eq 2.7). Modeled in this way, gear selectivity is interpreted as the proportion of age-*a* fish that will be caught by the purse-seine fishery per unit fishing mortality. Herring recruit into the model population at age-3 and this is the first age that is vulnerable to fishing in the model, therefore parameter *α*_*v*_, which is the age at which 50% selectivity occurs, was estimated using a uniform prior with bounds (3, 5) where the upper bound was chosen to yield a sufficiently wide interval. Parameter *β*_*v*_ is the slope at 50% selectivity, and a uniform prior was assigned to this parameter with reasonably broad bounds (1, 7).

Herring in Prince William Sound first spawn between the ages of three and five [5,35]. Therefore, maturity proportions for age-3 and age-4 herring were estimated, in lieu of maturity-at-age data, using the assumption that age-5 herring were fully mature (Eq 3.17–3.20). Previous studies indicate that maturity schedules for age-3 and age-4 herring in PWS changed after 1997 [17], thus different sets of maturity parameters were estimated for the two periods. The proportion of age-3 fish that are mature is forced to be less than the proportion of age-4 fish from the same brood year. Specifically, the maturity of age-3 fish is estimated as a fraction of the age-4 maturity parameter that is bounded between (0.00, 0.75) (Eq 3.17). The proportion of age-4 herring that are mature after 1997 was held constant at 0.9 to ensure model convergence; without this assumption, this parameter was stuck at its upper bound, affecting differentiability (Eq 3.20). These maturity parameters and empirical weight-at-age relationships were used to transform total abundance (Eq 2.8–2.9) into spawning biomass (Eq 2.12–2.13).

### Model formulation: Likelihood components and expressions

The original ASA model minimized the sum of squares for the fit of the model to the data. In the updated model, we use likelihoods so that statistical weights can be assigned to each dataset automatically. Six likelihoods relate model estimates to the observed age-compositions from the purse-seine catch and herring-spawn survey data, egg deposition estimates, two hydroacoustic biomass indices, and mile-days of milt index (Table 5).

**Table 5:** Components contributing to the negative of the logarithm of the likelihood expression for the Bayesian ASA model.

No	Likelihood component	Form
5.1	Complete expression	$L=\sum_{i=1}^{6}L_{i}$
5.2	Purse-seine age-composition	$L_{1}=-\sum_{y=1980,\ldots }^{∼1998}\left[Z_{1,y}\sum_{a\in A}\Theta_{1,y,a}ln(\frac{{\hat{\Theta }}_{1,y,a}}{\Theta_{1,y,a}})\right]$
5.3	Spawner survey age-composition	$L_{2}=-\sum_{y=1982}^{2012}\left[Z_{Sp,y}\sum_{a\in A}\Theta_{Sp,y,a}ln(\frac{{\hat{\Theta }}_{Sp,y,a}}{\Theta_{Sp,y,a}})\right]$
5.4	Number of eggs deposited	$L_{3}=10\sum_{y\in Y_{E}}\left[\ln\left(\sigma_{E,y}\right)+\frac{{\left(\ln\left({\hat{E}}_{y}\right)-\ln\left(E_{y}\right)\right)}^{2}}{2\sigma_{E,y}^{2}}\right]$
5.5	Total variance for L_3_	$\sigma_{E,y}^{2}=\sigma_{E,A,y}^{2}+\sigma_{E,B}^{2}$
5.6	ADF&G hydroacoustic biomass	$L_{4}=5\ln\left(\sigma_{H_{1}}\right)+\frac{1}{2\sigma_{H_{1}}^{2}}\sum_{y\in Y_{H}}{\left[\ln\left({\hat{H}}_{1,y}\right)-\ln\left(H_{1,y}\right)\right]}^{2}$
5.7	PWSSC hydroacoustic biomass	$L_{5}=20\sum_{y\in Y_{H}}\left[\ln\left(\sigma_{H_{2},y}\right)+\frac{{\left(\ln\left({\hat{H}}_{2,y}\right)-\ln\left(H_{2,y}\right)\right)}^{2}}{2\sigma_{H_{2},y}^{2}}\right]$
5.8	Total variance for L_5_	$\sigma_{H_{2},y}^{2}=\sigma_{H_{2},A,y}^{2}+\sigma_{H_{2},B}^{2}$
5.9	Milt mile-days	$L_{6}=33\ln{(\sigma }_{T}^{})+\frac{1}{2\sigma_{T}^{2}}\sum_{y\in Y_{}}{\left[ln({\hat{T}}_{y})-ln(T_{y})\right]}^{2}$

The age-compositions from the purse-seine fishery and the ADF&G herring-spawn survey were estimated from gear-selectivity and the ratio of numbers-at-age in year *y* to total numbers in year *y* (Eq 2.19–2.20). A multinomial distribution was assumed for the proportions-at-age from the purse-seine fishery and the ADF&G herring-spawn survey age-compositions (5.2–5.3). Due to schooling behavior of herring and gear-selectivity, original survey sample sizes need to be decreased to levels that reflect the actual variance contained in the sample. The effective sample size for each series *i* for year *y* ($Z_{i,y}^{\prime }$) was estimated using a modified version of the iterative reweighting procedure [37]:

$${Z^{\prime }}_{i,y}=\sum_{a=3}^{9+}\frac{{\hat{\Theta }}_{i,y,a}^{}(1-{\hat{\Theta }}_{i,y,a}^{})}{{\left(\Theta_{i,y,a}^{}–{\hat{\Theta }}_{i,y,a}^{}\right)}^{2}},$$

which takes advantage of a ratio of observed (*Θ*_*i*,*y*,*a*_) to estimated age-compositions ${\hat{\Theta }}_{i,y,a}$ from series *i* across years and iteratively estimates sample sizes until the process converges to a final set. This set of effective sample sizes was then supplied to the ASA model as input for the MCMC runs (see below). We modified this approach by using the harmonic mean (across years) of the ratio of estimated to original sample size in each iteration to reduce the input sample size used in the next iteration of the reweighting algorithm, as recommended by Stewart and Hamel [38].

Naturally spawned eggs were estimated from data on the numbers of eggs per female of age *a* multiplied by the numbers-at-age of female spawners for year *y* given by the post-fishery spawning abundance estimates and proportion-female data (Eq 2.21). A key assumption was that the egg deposition data were used as an absolute index of abundance. Predictions were tuned to observed egg deposition using the assumption of log-normally distributed errors (Eq 5.4) with annual coefficient of variation (CV) of *σ*_*E*,*y*_ (Eq 5.5).

The egg deposition data and the PWSSC hydroacoustic data include sample annual 95% confidence intervals. The methods outlined in Buckland [39] were used to derive sample CVs for the data (Eq 1.13 and 1.16), using the assumption that the CV of a log-normally distributed random variable asymptotically approaches the standard deviation of the logarithm of that variable. The model utilizes these survey-derived CVs, along with estimated additional error (Eq 3.13–3.14), to characterize total uncertainty (Eq 5.5–5.8).

Model biomass was multiplied by an estimated scaling factor, *q*_1_, to compare it to the ADF&G hydroacoustic biomass estimates (Eq 2.17) of mature and immature herring of ages 3 and older in the Sound before the spring catches are removed. This scaling factor *q*_1_ was estimated using a broad, uniform prior (Eq 3.10), and a lognormal distribution was assumed for the sampling distribution for the hydroacoustic data (Eq 5.6). Since no estimates of sample variance were provided, total uncertainty in the acoustic biomass was estimated as a year-independent CV (Eq 3.11) to represent model, process, and observed variance [40].

Model biomass was multiplied by a separate estimated scaling factor, *q*_2_, to compare it to the PWSSC hydroacoustic biomass estimates (Eq 2.18). The scaling factor *q*_2_ was estimated using a log-link and a broad, uniform prior (Eq 3.12) under the assumption of log-normally distributed measurement errors (Eq 5.7). As in the egg deposition component (discussed above), survey-derived sample CVs were used, along with estimated additional error (Eq 3.13), to characterize total uncertainty (Eq 5.8).

Predicted mile-days of milt were estimated using the ratio of male post-fishery spawning biomass to the parameter *q*_*T*_, which represents the tonnage of male biomass required to produce a mile-day of herring spawn in log-space (Eq 3.15). A lognormal likelihood was assumed for the model fits to the milt index (Eq 5.9), and the bounds for the *q*_*t*_ parameter were chosen to be sufficiently wide as to effectively implement an uninformative prior in log-space. As with the ADF&G acoustic estimates, the CV for milt was estimated (Eq 3.16) since no estimates of sample variance were provided.

To perform a comparison of variances between the current ADF&G and the Bayesian models, the assumed values used by ADF&G to weight each sum of squares term for the three indices of biomass were used to derive the implied lognormal standard errors that would result in the assumed weights [40]. To further facilitate comparison of the assumed variance in the egg deposition data between the two models, the median across survey years of the total egg deposition CV (Eq 5.5) was used in the comparison.

### Projected pre-fishery run biomass in the next year of the model

For every saved set of parameters from the MCMC chain, pre-fishery run biomass was projected for the next year of the model, 2013. This biomass forecast is the primary management metric used to regulate herring harvest rates in the Sound and refers to the spawning biomass at the start of the spawning season, which is the expected biomass available to the spring harvesters. We extended the population-at-age matrix by one year to obtain a projection of age-4 and older abundance in 2013 and used the mean log-recruitment from the previous 10 years to estimate projected recruitment. The choice of using the mean of recruitment under-estimates uncertainty in the forecast by ignoring variation about the mean, but the running average of recruitment over the latter twenty years of the modeling horizon (1992–2012) has low variability, so projection was robust to longer or shorter intervals (results not shown). This abundance-at-age projection was converted to biomass-at-age using the arithmetic mean weight of each cohort from the previous five years and the estimated maturity parameters.

### Implementation and convergence tests

The ASA model used by ADF&G is implemented in an Excel spreadsheet. The Bayesian ASA model used in this study employed the version of the Metropolis-Hastings MCMC sampler included in AD Model Builder [30,41] (Chib and Greenberg, 1995; Fournier et al., 2012) to construct a single chain of eleven-million iterations with a burn-in of 10%, thinning every thousandth sample. Convergence was reasonably accepted for all parameters using auto-correlation factor <0.10 and Geweke diagnostic statistic z <1.96, where the effective sample size for estimating the mean of each parameter across the MCMC chain, corrected for autocorrelation, was large enough to compare the Geweke statistic to the standard normal distribution.

### Sensitivity tests and retrospective analysis

A test of model sensitivity to the assumed value of 0.25 yr^-1^ for instantaneous, time-invariant background mortality of herring of ages 3–8 (Eq 3.1) was performed by running the Bayesian ASA Model using values of 0.15 yr^-1^ and 0.35 yr^-1^ for this parameter and comparing results. Evidence for a retrospective pattern was investigated by performing five retrospective runs of the Bayesian ASA Model starting in the same year (1980) and progressing for one fewer year each time [42]. In each retrospective run, the final year’s data are “peeled away” and the resulting forecast for year *n* using data ending in year *n-1* is compared to the model estimate of year *n* using data ending in year *n* to reveal systematic over or under-estimation by the assessment model. The degree of retrospective bias for the forecast biomass was quantified using Mohn’s *ρ*, which is the average relative difference (across retrospective “peels”) between the most recent estimate from a retrospective run and that from the “reference model” in the same year *B*_2013−*y*,ref_, which was the median pre-fishery run biomass from the current model using the entire data set [42,43]. Therefore, Mohn’s *ρ* for this study is defined as:

$$\rho =\bar{\left(\frac{B_{2013-y,p}-B_{2013-y,ref}}{B_{2013-y,ref}}\right)}$$

where the first subscript tracks the number of years into the past of the most recent estimate from the retrospective run of a given “peel” *p*.

## Results

### Comparison of data weighting

The data weighting values used by ADF&G, expressed as lognormal standard errors, are included in the 95% credible intervals for the acoustic and milt CVs (Table 6). The implied standard errors used in the ADF&G ASA model for the egg deposition and milt data are approximately 10% larger than the median estimated CV for these data types, but the implied standard error used by ADF&G is approximately 30% smaller than the median CV for milt estimated by the Bayesian model (Table 6).

**Table 6:** Comparison of weights between the ADF&G model and the Bayesian model. The first column lists the weights used in the ADF&G weighted least squares model (λ), the second column shows those weights converted to standard errors (σ) (Francis, 2011), and the third column shows the coefficients of variation (CV) used in the Bayesian model for the same data. Median and 95% posterior intervals are shown for the ADF&G hydroacoustic biomass and milt CVs. Sample errors were provided for the years that the egg-deposition survey ran (Eq 1.13), which were combined with the assumed value of additional error (0.40; Eq 3.14) to derive total egg deposition CV. Therefore, the median total CV across the survey years is presented for comparison.

	ADF&G model		Bayesian model
Data type	*λ*	*σ*	CV Median and 95% int.
Eggs deposited	0.25	0.45	0.43
ADF&G hydroacoustic survey biomass	0.50	0.32	0.29 (0.15, 0.55)
Milt	1.00	0.22	0.33 (0.25, 0.44)
PWSSC hydroacoustic survey biomass	-	-	0.35 (0.25, 0.53)

### Comparison of Bayesian posteriors to indices of biomass

This section compares posterior medians and 95% posterior predictive intervals to the observed means and 95% confidence intervals (CI) of each time series used in model fitting. The 95% CI for each time series were derived using the posterior median (if estimated) or assumed coefficients of variation (CV) listed in Table 3 [39].

Milt mile-days were well fitted by the Bayesian model given the 95% posterior predictive intervals (Fig 5A). The total estimated CV for the milt mile-days had a median and 95% credible interval of 0.33 (0.25, 0.44) (Table 6) and the estimated male biomass in 10^3^ mt required to produce a mile-day of herring spawn was 322.58 (95% interval 248.71–421.78) (Eq 3.15). The model estimate of egg deposition in 1989 was below the 95% posterior predictive interval, but the predictive intervals for the remaining years encompassed the data points and therefore were reasonably good (Fig 5B).

ADF&G acoustic biomass was predicted using total pre-fishery biomass multiplied by the estimated ADF&G acoustic scalar (Eq 3.10). The 95% posterior predictive intervals contained all five mean observations from the ADF&G acoustic biomass survey; meaning fits were reasonably good (Fig 5C). The 95% probability interval for the log-link scalar included zero (–0.72, 0.03), which translates to an interval of (0.49, 1.03) and a median of exp(−0.34) = 0.71 on the natural scale. Since the interval contains zero, the posterior includes the possibility that the ADF&G hydroacoustic survey is an unbiased estimate of the pre-fishery total biomass, but the median implies this survey is biased low, on average (Eq 2.11). The PWSSC acoustic biomass was estimated using total pre-fishery biomass multiplied by the estimated PWSSC acoustic scalar (Eq 3.12). The 95% posterior predictive intervals contained all twenty mean observations from the PWSSC hydroacoustic biomass survey (Fig 5D). The 95% probability interval for the log-link scalar, translated to the natural scale, is (0.56, 0.96) with a median of exp(−0.30) = 0.74. Hence, both hydroacoustic surveys, on average, underestimate the pre-fishery total biomass (Eq 2.11) and the PWSSC survey is more precise.

### Comparison of Bayesian posteriors to age-composition data, and recruitment

**Fig 6:**
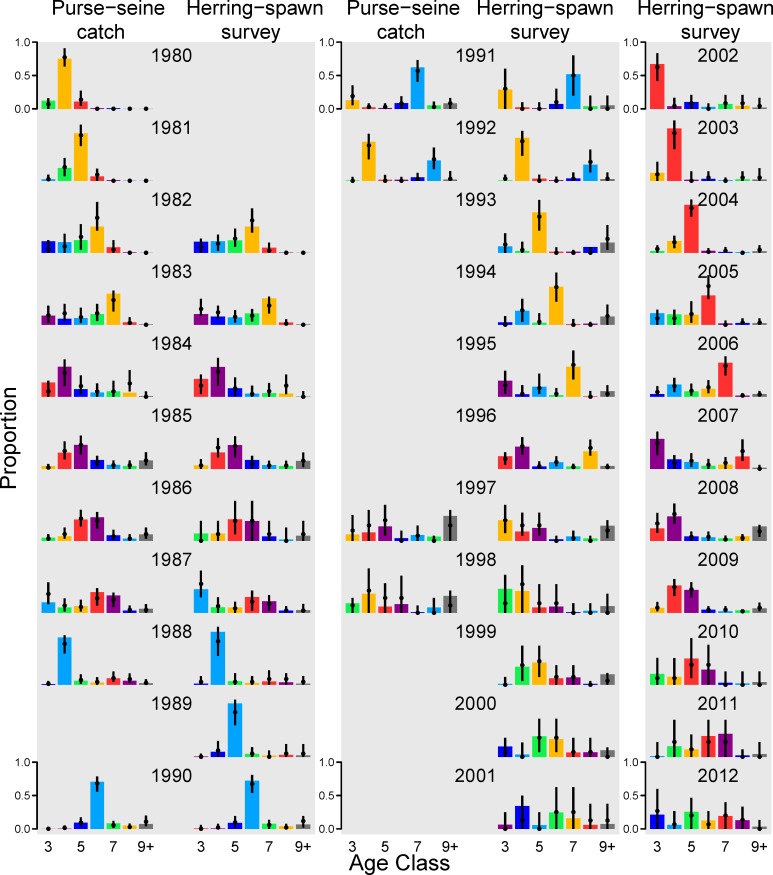
Model fits to the two sets of age-composition data used: proportion of catch-at-age from the purse-seine fishery and age-composition proportions from the ADF&G herring-spawn survey. Colored bars denote data, colors track individual cohorts through time, and points show posterior median with bars showing the 95% posterior intervals. No compositions are shown for years when the spring fishery was closed (1989, 1993–1996, and after 1998).

**Fig 7:**
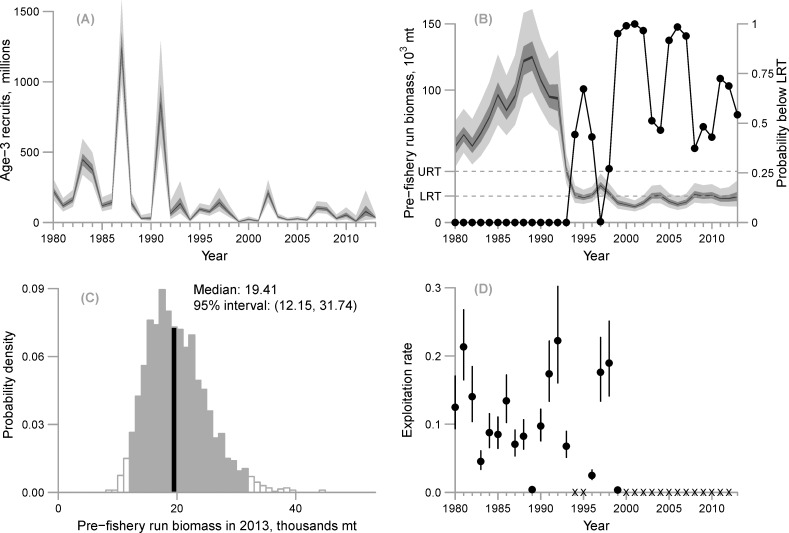
(A) Estimated recruitment at age-3 (posterior intervals; light gray = 95% interval, darker gray = 50% interval, black = 5% interval), (B) estimated pre-fishery biomass (posterior intervals; light gray = 95% interval, darker gray = 50% interval, black = 5% interval) and the probability that pre-fishery biomass is below the lower regulatory threshold (LRT) of 22,000 short tons (19,958 mt) (connected black points) with the upper regulatory threshold (URT: 42,500 short tons, 38,555 mt) shown for reference, (C) posterior distribution of estimated pre-fishery biomass for 2013 with the 95% credible interval (light grey) and the median (black) shown, and (D) posterior median exploitation rates (black points) with 95% posterior intervals (segments)—“X” characters represent years the fishery was closed.

Strong cohort signals, which can be clearly identified across consecutive years, are present and consistent in both sets of estimated age-compositions (Fig 6). The largest estimated cohorts were spawned in 1976, 1980, 1984, 1988, and 1999, and clearly match the strongest cohort signals identified in the seine and herring-spawn age-composition data (Fig 6) for cohorts born in 1980, 1984, 1988, and 1999. Similar signals are seen in the estimates of age-3 fish (Fig 7A), where the largest events occurred in 1983 (median of 446 million fish), 1987 (median of 1,234 million fish), and 1991 (median of 840 million fish) and the smallest recruitment events occurred in 1999 and 2011 with estimated median recruitment of fewer than 10 million fish (Table 4).

### Pre-fishery run biomass

The population collapse of 1992–93 is evident in the estimated biomass trajectory where the highest estimates in the post-collapse period are less than the lowest estimated biomass from the pre-collapse period (Fig 7B). Biomass was highest in 1988–89 (median and 95% intervals of 120,750 (93,920, 161,130) and 124,900 (98,310, 162,760), respectively), and lowest from 1999–2002 (all 95% intervals in this period are contained by a range of 8,740 mt to 20,640 mt) and again from 2005–7 (all 95% intervals contained within 9,570 mt and 20,540 mt). The posterior median of the final year biomass (2013) was estimated to be 19,410 mt with a 95% credible interval of (12,150 mt, 31,740 mt) (Fig 7C, Table 4).

### What is the probability that biomass will fall below the lower regulatory threshold?

There was a 0.54 probability that the biomass forecast in 2013 was below the lower regulatory threshold of 19,555 mt (Table 4). The probability was zero that pre-fishery run biomass was below the lower regulatory threshold from 1980–93 (Table 4; Fig 7B). For the post-collapse period, there was zero probability that biomass in 1997 was below the lower regulatory threshold, which corresponds to a sharp increase in both the PWSSC acoustic biomass (observed value of 37,400 mt) and the milt index (observed value of 64.30 mile-days) in that year. Conversely, the probability that herring biomass was low enough to warrant closure of the commercial fishery was >0.90 in 1999–2007, except for 2003–4.

### Exploitation rate

Exploitation rate was defined as total catch (converted to mt using the empirical weight-at-age matrix, Eq 1.7) divided by the 95% credible intervals for pre-fishery run biomass. Median exploitation for all years was estimated to be less than or equal to 0.22, and the highest median exploitation rates occurred in 1981 and 1992 (Fig 7D). In 1989, 1996, and 1999, access was limited to either the food/bait or spawn-on-kelp fisheries; therefore the lowest exploitation rate resulting from a full commercial harvest occurred in 1983 (median 0.05, 95% interval 0.03–0.06) (Table 4).

### Selectivity and life history parameters

**Fig 8:**
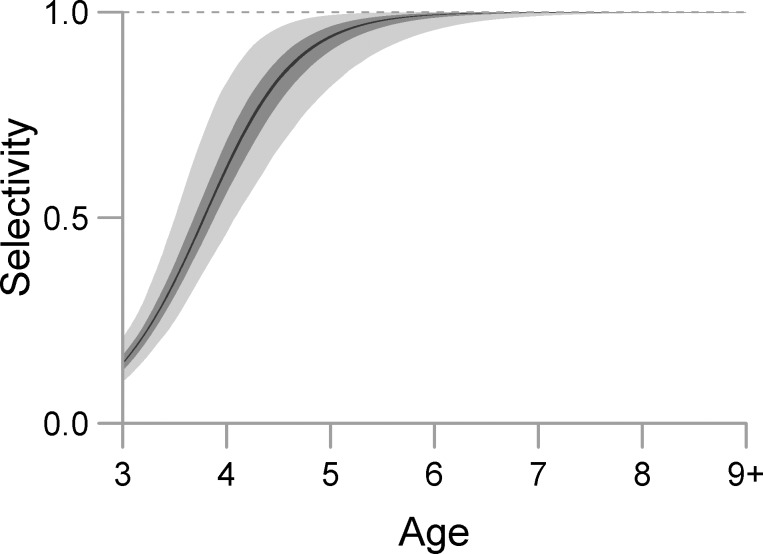
Posterior distribution (light gray = 2.5–97.5% interval, darker gray = 25–75% interval, black = median) for model estimates of selectivity proportion at age.

**Fig 9:**
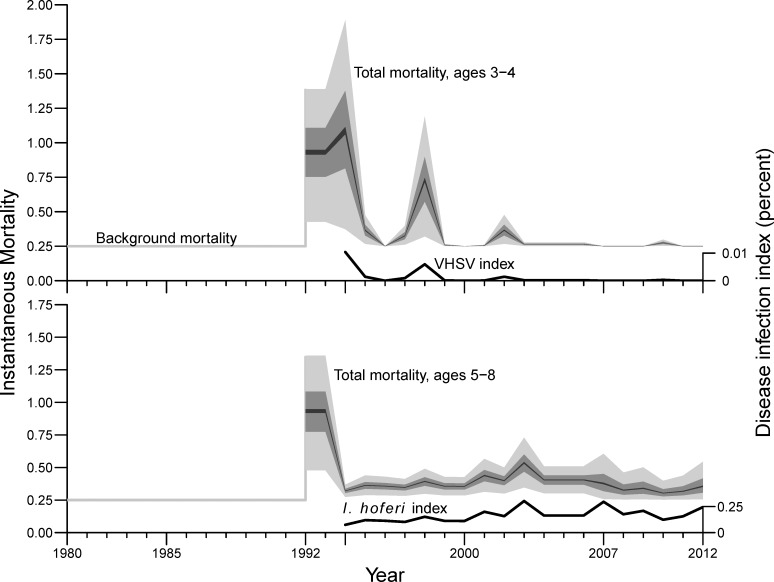
Total non-fishery mortality rates for herring of ages 3–8 in 1980–1991 (assumed) and in 1992–2012 (estimated). Posterior distributions begin in 1992 (light gray = 95% interval, darker gray = 50% interval, black = 5% interval) for model estimates of total non-fishery instantaneous mortality for ages 3–4 (top) and ages 5–8 (bottom). Lower black lines show the disease index data for VHSV and *I*. *hoferi* infection (percent; right-hand axis). VHSV infection was observed to be zero in 2007–2009 and 2011–2012, hence total instantaneous mortality in those years equals the assumed background level of 0.25 yr-1.

The Bayesian model provided estimates of the age and instantaneous rate of change at 50% selectivity using purse-seine gear, and proportions of fish at ages 3 and 4 that are mature in the first and second regime (Eq 3.8–9 and 3.17–20; Fig 8). Multiple parameters used to model total instantaneous mortality for herring of ages 3–9^+^ were also estimated (Eq 3.2–3.7). Total instantaneous mortality (including additional mortality from VHSV) for herring of ages 3–4 was highest in 1992–1994 and in 1998, and was below 0.40 yr^-1^ in the remaining years (Table 4; Fig 9). The rate of total instantaneous mortality (including additional mortality from *I*. *hoferi)* for herring of ages 5–8 was highest in 1992–1993 and 2003 (Table 4; Fig 9), and was below 0.50 yr^-1^ for the remaining years (Table 4; Fig 9).

### Sensitivity analysis and retrospective pattern

**Fig 10:**
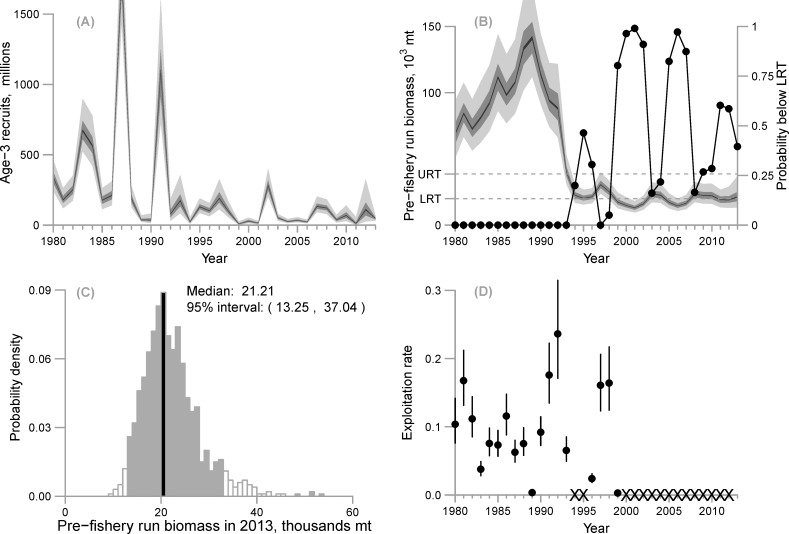
Results from the sensitivity analysis using the fixed value of 0.35 yr-1 for background natural mortality. See Fig 7 caption for explanation of panels, colors, and symbols.

**Fig 11:**
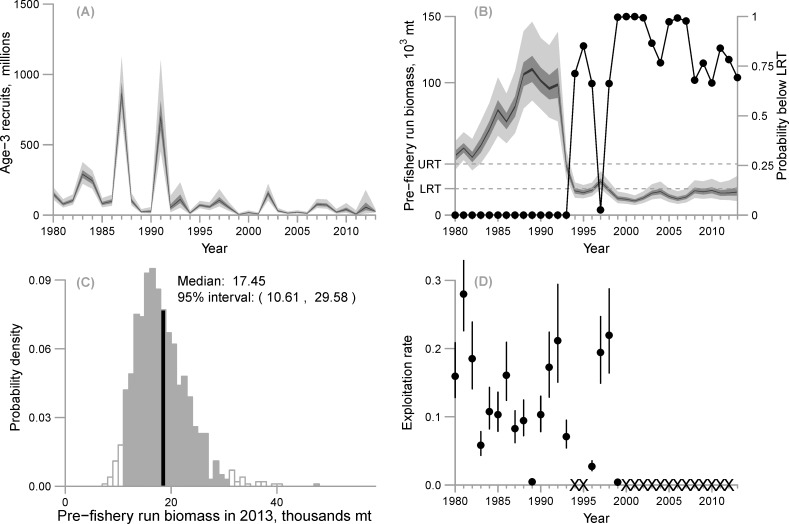
Results from the sensitivity analysis using the fixed value of 0.15 yr-1 for background natural mortality. See Fig 7 caption for explanation of panels, colors, and symbols.

**Fig 12:**
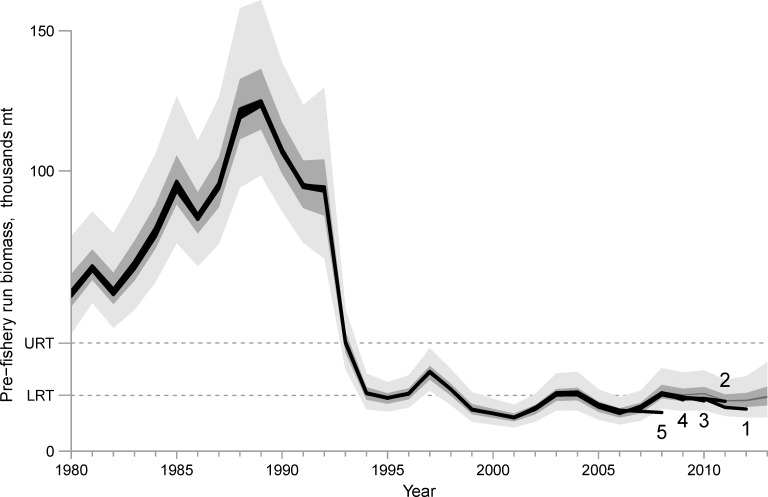
Results from five retrospective “peels” compared to the posterior intervals (light gray = 95% interval, darker gray = 50% interval, black = 5% interval) of pre-fishery run biomass from the Bayesian model fit to the entire time series of data. Each “peel” is the posterior median of the model run with an additional year of data removed and is labeled numerically where a higher number denotes a peel of data further into the past.

Increasing the fixed rate of background mortality (Eq 3.1) from the assumed value of 0.25 yr^-1^ to 0.35 yr^-1^ led to an increase in the scale of recruitment for all years, but also resulted in an increase in the scale of the estimated biomass for all years such that the median estimated 2013 forecast biomass was approximately 2,000 mt greater than that using 0.25 yr^-1^ (median 21,210, 95% interval 13,250–37,040) (Fig 10). Decreasing the rate of background mortality to 0.15 yr^-1^ led to a decrease in the scale of recruitment for all years and resulted in a decrease in the scale of the estimated biomass for all years such that the median estimated 2013 forecast biomass was approximately 2,000 mt less (median 17,450, 95% interval 10,610–29,580) (Fig 11). The retrospective analysis revealed systematic underestimation in the posterior medians of pre-fishery run biomass in the most recent five years compared to the ASA Model using the entire duration of available data (Fig 12) and the average Mohn’s *ρ* for these peels was -0.15.

## Discussion

This study demonstrated several benefits of adopting the Bayesian version of the PWS herring ASA Model, namely the ability to work with a conceptually simple definition of uncertainty via credible intervals, and the assignment of probabilities associated with alternative states of the population, such as the chance pre-fishery run biomass is below the lower regulatory threshold, which are useful for risk analysis. Furthermore, the Bayesian ASA model lacks evidence of a retrospective pattern in the forecast, but is sensitive to assumed values of baseline non-fishery mortality.

### Comparison of Bayesian estimates of pre-fishery run biomass and the current ADF&G model

ADF&G’s forecast for 2013 was 23,673 mt, which is above the lower regulatory threshold of 19,958 mt. The ADF&G estimate is within the 95% credibility interval from the Bayesian model (12,150 mt, 31,740 mt) (Fig 5C; Table 4), and 22% higher than the Bayesian posterior median of 19,410 mt. The principal structural difference between the model developed in this study and ADF&G’s current herring model is the way uncertainty is modeled and incorporated into the estimation process, and the statistical basis in the Bayesian model for weighting of different data sources. One of the most important reasons to conduct fisheries stock assessment is to be able to evaluate the consequences of alternative management actions [27]. Therefore, one compelling reason to adopt the Bayesian model is to facilitate this type of management decision analysis using objective and intuitive “weights” of population states under different management scenarios in the form of posterior probabilities.

The transformed ADF&G weights, expressed as lognormal standard errors, were similar to the medians from the Bayesian model for ADF&G acoustic biomass and the egg deposition CVs. However, the 95% intervals for the CV of the ADF&G acoustic biomass estimates include values that are half that and close to double the assumed ADF&G error. Furthermore, the median CV on milt estimated by the Bayesian model is approximately 50% higher than the assumed ADF&G value, so the Bayesian model does involve more uncertainty, on average, in the milt data than the weighted least squares model.

### Benefits and implications of using a Bayesian PWS herring assessment model

Adopting a Bayesian assessment model could require a revision of the regulations used by ADF&G to manage the PWS herring population. As previously mentioned, ADF&G compares the point estimate forecast of pre-fishery run biomass from their weighted least squares assessment model to the lower and upper regulatory thresholds to set the season’s harvest rate [44]. However, the Bayesian forecast, in the form of a probability distribution of pre-fishery run biomass, introduces important management questions: Should the median or the mean of the distribution be used in place of the point estimate, and how should uncertainty in the forecast in the form of credible intervals be used to set harvest rates?

Regulations that compare only a measure of central tendency (the mean, median, or mode) of the posterior distribution to a limit do not utilize the complete Bayesian model output. The mean and mode of the posterior distribution of a management metric, such as the forecast of biomass, may fall into any interval of the distribution leading to regulations that have shifting associated probabilities from year to year. The posterior median represents the 50^th^ percentile of the distribution, and is therefore robust to the aforementioned limitation, but as with the previous two measures, the median contains no information on the variance associated with the forecast biomass.

Probabilistic decision rules that take into account the posterior distribution of forecast biomass are possible with a Bayesian assessment model, as for example outlined in Shertzer et al. [45]. A menagerie of stochastic assessment regulations exist and there are methods to help managers determine or design the best performing rule given the specific ecological, economic, or conservation objectives tied to the status of their stock. One possible type of decision rule may require a specific minimum probability that the stock is above a certain limit for fishing to continue. For example, Kurota et al. [46] outline how southern bluefin tuna is managed by requiring a 70% probability that the biomass is above a threshold value. Hypothetically, a more conservative approach would require a 90% probability, while a riskier regulation would use a 30% probability. The modifiers “conservative” and “risky” in this example only refer to the increase or decrease in associated probability of the true biomass being above the regulatory limit, respectively.

Determining which credible intervals or cumulative probabilities are best for managing PWS herring (60% or 75% or 90%, etc.) involves trade-offs with respect to conservation goals and ecosystem balance, as well as economic concerns, and any recommendations should be made after conducting a risk analysis and/or management strategy evaluation (MSE) using the goals of the agency and the community. In the interim between adopting the Bayesian ASA Model and completing an MSE, a decision rule may need to be adopted and, given the particular importance of this population, our recommendation would be to adopt a conservative rule that requires an estimated 100% probability that the stock is above the lower threshold of 19,958 mt for two consecutive years for a limited harvest rate to be set. This recommendation first assumes that the lower limit adopted by ADF&G in 1995 [20] continues to represent the amount of biomass required to maintain the herring stock going forward. Furthermore, it considers the variability of herring recruitment from year to year and assumes that if herring biomass was sufficiently high last year to satisfy the opportunistic needs of the ecosystem and remain above the limit this year, then there is enough to sustain a limited catch in the current year. Managing this population using a fully risk-averse rule (0% probability that biomass is below the limit) relies on the ability of the Bayesian model to integrate over all included sources of uncertainty, but admits the possibility that other driving factors, not yet integrated into the model, may impact the variability of herring biomass and recommends that managers be as cautious as possible.

Other management agencies also use regulatory rules that extend beyond a single year. For example, the maximum constant yield (MCY) used in New Zealand states that no management regulation should allow the stock to drop below 20% of virgin biomass over a specific time horizon with greater than 10% probability [29]. Therefore, in addition to the recommendation to test for appropriate widths of credible intervals, a management strategy evaluation comparing regulation rules involving a combination of risk over a range of horizons would also provide vital information for PWS herring management going forward.

### Model sensitivities

The conflict between the milt and egg deposition data is in the years 1988–89 and 1991–92 (Fig 5) and it is in these years that the fit to the milt and egg deposition data are degraded–this is because the model splits the differences between the conflicting trends in the egg deposition and milt data by minimizing errors to both fits. The result is that milt is underestimated when egg deposition is overestimated in 1988 and vice-versa in 1991.

Previous research on the PWS herring assessment model involved a sensitivity analysis using time-invariant instantaneous mortality rates between (0.35 yr^-1^, 0.55 yr^-1^) and concluded the main effect of higher mortality is larger recruitment and vice-versa [5]. This effect occurs because a population that experiences a higher rate of mortality would need to have larger recruitment cohorts to maintain the biomass levels observed on the surveys. The sensitivity analysis conducted here on time-invariant background mortality (Eq 3.1) reveals a similar correlation between mortality and the scale of the recruitment estimates; higher baseline mortality results in larger recruitment in all years and lower mortality results in lower recruits, on average. However, varying the assumed value for background mortality also had an impact on the biomass forecast; a background mortality rate of 0.35 yr^-1^ would result in four median biomass estimates out of the last six years of the modeling horizon being above 20,000 mt (Fig 9). An important next step would be to explore the sensitivity of the agency’s regulatory threshold to the assumed rate of non-fishery mortality.

We used the guidelines suggested by Hurtado-Ferro et al. [43] to contextualize the results from the retrospective analysis, which state for a short-lived, sardine-like life history, a Mohn’s *ρ* outside of the interval (-0.22, 0.30) would be of concern, but a Mohn’s *ρ* within the interval may or may not be of concern and may need further investigation. The magnitude and direction of Mohn’s *ρ* is not related to that of the bias in final-year biomass compared to the original assessment, but it may be related to the direction of some recent, true change in the population with respect to some time-invariant parameter of the model [43]. Recent changes in mortality, growth, or selectivity have been identified as potential drivers behind a retrospective pattern [47] and a positive retrospective pattern is of more conservation concern than a negative pattern. Therefore, the results for Prince William Sound herring (Mohn’s *ρ* = -0.15) may give evidence of a recent decrease in true background mortality rate or true selectivity. However, the retrospective pattern of the first five peels are less than the model-estimated uncertainty in biomass (Fig 12), and so this study reveals a slight, negative retrospective pattern that is of little concern.

### Next steps to improve the Bayesian PWS herring ASA model

Future research on this stock and from other herring stocks can be straightforwardly integrated into the Bayesian model in the form of informative, or more informative, priors. Candidate parameters that would benefit from informative priors are recruitment, maturity at age proportions, and background mortality. Other functional forms of the prior distributions can also be investigated, including using an inverse-gamma distribution for the coefficients of variation. The sensitivity tests performed here should be extended to include testing the impact of the assumed values for the proportion of impounded fish that die and the implied scalar for egg deposition data, which currently has a value of 1.00.

## Conclusions

We developed the first Bayesian assessment of Prince William Sound herring and found that the population remains at low levels, with a 54% probability that 2013 biomass is below the regulatory limit of 19,958 mt. Future work could use the model to assess which factors are behind this stock’s continued low abundance. The Bayesian model produces estimated quantities with measures of uncertainty and probabilities associated with alternative states of the population, which are useful for hypothesis testing. Furthermore, the Bayesian structure for the ASA model allows for the inclusion of further information on this stock as well as information from other herring stocks, in the form of informative, or more informative, priors.

Adoption of the Bayesian model as the agency’s assessment tool could also be the basis for managing this population with a decision rule that explicitly considers uncertainty. The amount of risk managers are willing to assume, in terms of strategic economic or conservation goals, can be built directly into a probabilistic regulation rule. Trade-offs with respect to conservation goals and ecosystem balance, as well as economic concerns, and any recommendations should be made after conducting a risk analysis and/or management strategy evaluation using the goals of the agency and the community.

## Supporting information

S1 Appendix Bayesian model code and data files for Prince William Sound herring stock assessment. (ZIP)
